# Does academic assessment system type affect levels of academic stress in medical students? A cross-sectional study from Pakistan

**DOI:** 10.3402/meo.v20.27706

**Published:** 2015-06-24

**Authors:** Madiha Ali, Hamna Asim, Ahmed Iqbal Edhi, Muhammad Daniyal Hashmi, Muhammad Shahjahan Khan, Farah Naz, Kanza Noor Qaiser, Sidra Masud Qureshi, Mohammad Faizan Zahid, Imtiaz Jehan

**Affiliations:** 1 Medical Graduate, Aga Khan University, Karachi, Pakistan; 2 Department of Community Health Sciences, Aga Khan University, Karachi, Pakistan

**Keywords:** assessment system, academic stress, medical education, medical students, stress

## Abstract

**Introduction:**

Stress among medical students induced by academic pressures is on the rise among the student population in Pakistan and other parts of the world. Our study examined the relationship between two different systems employed to assess academic performance and the levels of stress among students at two different medical schools in Karachi, Pakistan.

**Methods:**

A sample consisting of 387 medical students enrolled in pre-clinical years was taken from two universities, one employing the semester examination system with grade point average (GPA) scores (a tiered system) and the other employing an annual examination system with only pass/fail grading. A pre-designed, self-administered questionnaire was distributed. Test anxiety levels were assessed by The Westside Test Anxiety Scale (WTAS). Overall stress was evaluated using the Perceived Stress Scale (PSS).

**Results:**

There were 82 males and 301 females while four did not respond to the gender question. The mean age of the entire cohort was 19.7±1.0 years. A total of 98 participants were from the pass/fail assessment system while 289 were from the GPA system. There was a higher proportion of females in the GPA system (85% vs. 59%; *p*<0.01). Students in the pass/fail assessment system had a lower score on the WTAS (2.4±0.8 vs. 2.8±0.7; *p*=0.01) and the PSS (17.0±6.7 vs. 20.3±6.8; *p*<0.01), indicating lower levels of test anxiety and overall stress than in students enrolled in the GPA assessment system. More students in the pass/fail system were satisfied with their performance than those in the GPA system.

**Conclusion:**

Based on the present study, we suggest governing bodies to revise and employ a uniform assessment system for all the medical colleges to improve student academic performance and at the same time reduce stress levels. Our results indicate that the pass/fail assessment system accomplishes these objectives.

Stress and its implications on the physical and mental health and other aspects of life is a widely explored topic in scientific and academic research. Generally, academic stress inculcates a sense of competition and motivation among students and promotes learning. However, sometimes this stress produces anxiety and feelings of helplessness, leading to stress-related disorders and adversely affecting performance, academic and non-academic (1). As a consequence of academic pressure, students have been reported to resort to academic misconduct and even substance abuse as a coping method. Some withdraw from their studies altogether, unable to cope with the stress, while others even attempt/commit suicide (2–8).

‘Academic stress’ is a term extensively explained in literature and is used to describe the prevalence and implications of study-related stress among students and the negative impact it has on their performance (9–11). It has often been used interchangeably with ‘examination-related stress’ and it is hard to determine how much the two domains overlap. Since examinations are an integral part of the academic process, examination-related stress is regarded as a subtype of academic stress and used as a tool for assessment of the latter (9, 10, 12, 13).

Medical students are continuously challenged by comprehensive curricula and assessments against a backdrop of a highly competitive environment (14). Gupta et al. (15) defined ‘academic stress’ as a mental distress resulting from apprehension and frustration associated with academic failure, apprehension of such failure, or even an awareness of the possibility of such failure. Dahlin et al. (16) from Sweden quoted the prevalence of stress to be as high as 12.9% and a suicide attempt rate of up to 2.7% in medical students. A study investigating stress and worry in Thai medical students showed the prevalence at a staggering 61.4% (17). A similar study recently conducted in Lahore, Pakistan, stated the prevalence of stress as high as 20.8% among medical students, strongly associated with poor academic performance (18). Several other student population-based studies have shown high levels of stress among medical students (19–22), and some studies have even demonstrated high levels of stress in medical students in comparison to undergraduate non-medical students (16, 23, 24).

In Pakistan, the medical curriculum framework is formulated and standardised by the Higher Education Commission. This framework is continuously revised, improved, and implemented across the country to maintain uniformity and synchronisation among all higher education institutions. A modular system-based curriculum is currently recommended and widely followed by the majority of medical institutes (25).

Within the modular curriculum, there are two widely accepted assessment methodologies by the medical colleges. One of these employs a semester system with six-monthly examinations, graded with a four interval grade point average (GPA) tool, which is similar to other grade-tiered academic assessment systems. The other system is based on annual examinations, with the performance of students graded as either pass or fail (26).

Several medical schools are currently undergoing curricular reform. When considering the means by which students are evaluated in a revised curriculum, the need to reduce the prevalence of depression and anxiety associated with academic stress must be weighed against the importance of academic outcomes. To the best of our knowledge, no study comparing the levels of stress in different assessment systems has yet been conducted in Pakistan. We conducted this cross-sectional study to determine and compare the levels of stress among undergraduate medical students studying under the most commonly employed assessment systems (GPA vs. pass/fail) in order to identify the system that best assesses student performance while keeping stress levels to a minimum.

## Materials and methods

### Operational definitions

- Stress: Stress refers to experiencing events that are perceived as endangering one's physical or psychological well-being and thereby tax one's coping abilities. These events are usually referred to as stressors, and people's reactions to them are termed as stress responses.
- Academic stress: Academic stress is a mental distress with respect to some apprehension and frustration associated with academic failure, apprehension of such failure, or even an awareness of the possibility of such failure (15).
- Anxiety: Anxiety is a state of apprehension, tension, and worry. It can be distinguished from fear by the characteristic that the object of anxiety is less specific than the object of a fear.
- GPA system: Semester system with six-monthly examinations graded with a four interval GPA system.
- Pass/fail system: Annual examination graded by pass, fail, or pass with honours.
- Details of the teaching and assessment systems are provided in Appendix 1.

### Study design, setting, and participants

This was a cross-sectional study involving medical students of pre-clinical years (year 1 and year 2) enrolled at two medical colleges employing different assessment systems.

Two medical colleges were sampled for assessing the impact of different assessment systems on the levels of academic stress among medical students. Of the two institutions under study, the Aga Khan University Medical College follows the pass/fail assessment system. The second institution, the Jinnah Sindh Medical University, follows the GPA assessment system. A self-administered questionnaire was used for data collection.

The study was carried out in February 2013. In the GPA academic calendar, the students had already taken their half-yearly examinations in the preceding December 2012, with no subsequent examinations/assessments to approach shortly before June/July 2013. In the pass/fail calendar, the students had appeared for their short-term summative examinations in preceding January 2013, with no upcoming examinations before April 2013. These accounted for adequate and acceptable intervals between exams already taken, participation in the present study, and any future exams to be taken to have minimal effects, if any, on student responses.

### Data collection procedure

The study population was selected by convenience sampling. All participants were informed of confidentiality, anonymity, and the choice to refuse to participate in the study. A brief explanation of academic stress was given to each participant, after which those who agreed to participate in the study provided written and verbal informed consent and filled the questionnaire. During the filling-in of the questionnaire, the investigating team helped clarify any ambiguities regarding the questions, when needed, without influencing the answers.

### Data collection tool

A pre-designed, paper-based questionnaire was used for gathering data for this study. The questionnaire comprised four sections. Details of each section are as follows:

- Section 1 – Demographic details, comprised personal information on variables such as age, gender, residential area, year at medical college, name of institute, GPA in last semester, and satisfaction with academic performance.
- Section 2 – The Westside Test Anxiety Scale (WTAS), a 10-item instrument designed to identify students with anxiety. It has been validated on a statistically appropriate sample size of students enrolled in medical universities of Karachi (6). The WTAS allowed us to quantify the level of examination-related anxiety in our study population. Each item was scored on a scale from 1 to 5. The cumulative sum of all responses was divided by 10 to give the Test Anxiety Score. The interpretation of sum of scores of WTAS is provided in Appendix 2.
- Section 3 – The Aga Khan University Anxiety and Depression scale (AKUADS) consisted of 13 questions for qualitative assessment of the effects of exam anxiety on physiological functions of the students. Each item was given a choice of ‘yes’ or ‘no’.
- Section 4 – The Perceived Stress Scale (PSS), a 10-item scale, is a psychometric instrument used to measure and assess the perception of stress. It has already been validated on a statistically appropriate sample size of students enrolled in a medical university in Lahore, Pakistan (27). The scoring guideline and interpretation of sum of scores of PSS are provided in Appendix 2.

The questionnaire was tested during a pilot phase on 30 pre-clinical medical students in a similar setting. The collected data were analysed and the subjects were asked to report any possible difficulties faced concerning the questionnaire. No difficulties were reported. Data from these 30 subjects were discarded and not included in the final study.

### Data entry and analysis

The data were collected and field editing was done by all investigators. Unfilled forms were discarded. The data were entered into EpiData 3.1. The data were then exported to SPSS version 19 for analysis. Descriptive statistics, including means, standard deviations, 95% confidence intervals, and frequency distributions for all important variables calculated. Chi-square test and independent sample t-test were used to evaluate differences in results of categorical and continuous variables, respectively, between the two groups. An alpha level of 0.05 was used as a criterion for significance.

### Ethical considerations

The study was reviewed and approved by the Ethics Review Committee at the Aga Khan University Medical College. The study design was also reviewed by the administration at Jinnah Sindh Medical University. Both institutions granted permission to conduct the study on their respective student populations. Verbal and written informed consent was taken prior to questionnaire administration. All questionnaires remained anonymous. The information gathered during this study was accessible only to the primary investigating team conducting the study. The informed consent forms were separated from the questionnaires prior to data entry to maintain anonymity participants.

## Results

A total of 400 questionnaires were distributed among pre-clinical medical students at both medical schools. Of the 400 questionnaires, 387 (97%) were completed and returned by 82 males and 301 females; four participants did not respond to the gender question. The mean age of the entire cohort of participants was 19.7±1.0 years. Fifty-four were first-year medical students and 331 were in their second year, while two participants did not mention their current year of study. Ninety-eight participants (25%) were from the pass/fail assessment system while 289 (75%) were from the GPA assessment system.

The demographic details of students in both assessment systems are displayed in Table 1. The mean age of the participants was similar at both institutions (19.5 vs. 19.8 years; *p*>0.1). There was a significantly higher proportion of female participants from the GPA system in comparison to pass/fail system (85% vs. 59%; *p*<0.01). More students were enrolled in their second year of medical college in the GPA system as compared to pass/fail system (91% vs. 70%; *p*<0.01). More students in the GPA system were only moderately or not satisfied with their academic performance than students in the pass/fail system, whereas more students in the pass/fail system said they were completely satisfied with their performance than those in the GPA system. The proportion of day scholars and hostelite students in both systems were comparable (*p*>0.1).

**Table 1:** Demographic details of students in different assessment systems

		Assessment system		
			
Characteristics		Pass/fail/honour system, *n* (%)	GPA system, *n* (%)	*p*
Total students		98 (25)	289 (75)	–
Age, years, mean±SD		19.5±0.9	19.8±1.0	0.322
Gender	Male	40 (41)	42 (15)	<0.01
	Female	58 (59)	243 (85)	
Year of study	1st year	29 (30)	27 (9)	<0.01
	2nd year	69 (70)	262 (91)	
Residence	Day scholar	64 (65)	167 (58)	0.190
	Hostelite	34 (35)	122 (42)	
Satisfaction with academic performance	Completely satisfied	27 (28)	35 (12)	<0.01
	Moderately satisfied	55 (56)	135 (47)	
	Not satisfied	16 (16)	119 (41)	

**Fig. 1:**
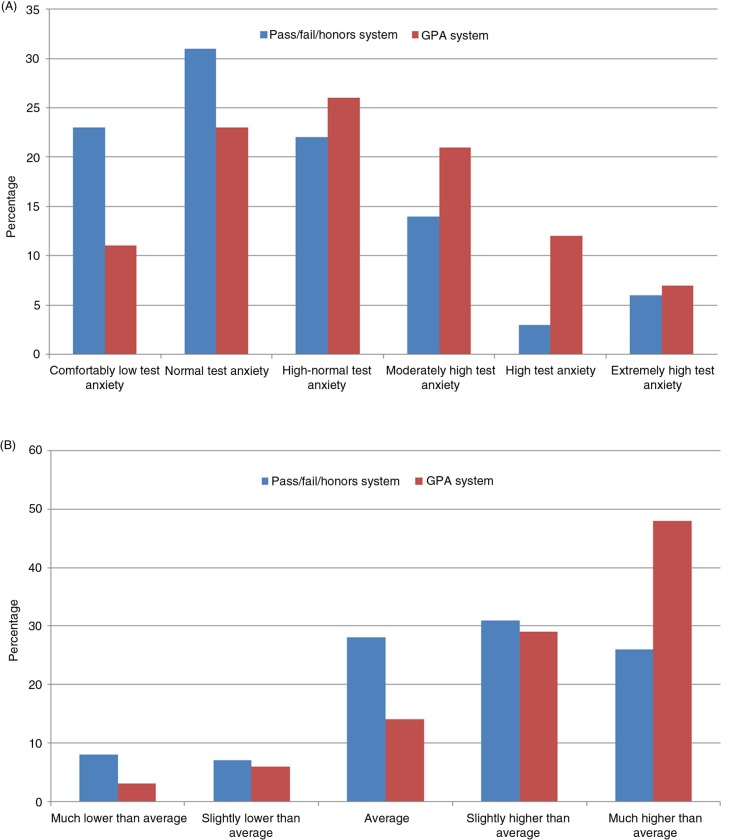
Comparison of the proportions of participants in both assessment systems falling in the different categories of the (A) Westside Test Anxiety Scale and (B) the Perceived Stress Scale.

Comparison of mean scores of the WTAS and PSS were statistically significant between the two assessment systems. Students in the pass/fail assessment system had a lower score on the WTAS (2.4±0.8 vs. 2.8±0.7; *p*=0.01) and the PSS (17.0±6.7 vs. 20.3±6.8; *p*<0.01), indicating lower levels of test anxiety and overall stress than in students enrolled in the GPA assessment system. A greater number of students in the pass/fail system scored towards the low side of both scales, whereas students in the GPA system scored towards the higher side of the scales (Table 2 and Fig. 1).

**Table 2:** Categorical comparison of Westside Test Anxiety Scale and Perceived Stress Scale between the two assessment systems

			Assessment system				
				
Category			Pass/fail system, *n* (%)		GPA system, *n* (%)		*p*
Total students			98		289		
Westside Test Anxiety Scale	Low – normal test anxiety	Comfortably low test anxiety	75 (77)	23 (24)	173 (60)	33 (11)	0.03
		Normal test anxiety		30 (31)		66 (23)	
		High–normal test anxiety		22 (22)		74 (26)	
	High test anxiety	Moderately high test anxiety	23 (23)	14 (14)	116 (40)	60 (21)	
		High test anxiety		3 (3)		36 (12)	
		Extremely high test anxiety		6 (6)		20 (7)	
Perceived Stress Scale	Low – average stress	Much lower than average	42 (43)	8 (8)	66 (23)	8 (3)	<0.01
		Slightly lower than average		7 (7)		18 (6)	
		Average		27 (28)		40 (14)	
	Above average stress	Slightly higher than average	56 (57)	30 (31)	223 (77)	85 (29)	
		Much higher than average		26 (26)		138 (48)	

Questions assessing the physical manifestations of stress were adapted from the AKUADS scale. Headache was reported as the most frequent symptom in both groups, with the prevalence being as high as 50% in the pass/fail system and 74% in the GPA system. Other commonly reported symptoms included tension in neck and shoulders (32.7% in pass/fail, 49.5% in the GPA system), reduced sleep (45.9% in pass/fail, 38.8% in the GPA system), nausea (34.7% in pass/fail, 31.8% in the GPA system), sensation of pain all over the body (20.4% in pass/fail, 42.9% in the GPA system), and loss of appetite (16.7% in pass/fail, 28.2% in the GPA System). From the 13 symptoms that were assessed, loss of appetite, headaches, sensation of tension in neck and shoulder pain all over the body were significantly different between the two systems (Table 3).

**Table 3:** Physical manifestations of stress in students of the two systems

	Assessment system		
		
Variable	Pass/fail system, *n* (%)	GPA system, *n* (%)	*p*
Total students	98	289	–
Headaches	49 (50)	214 (74)	<0.01
Sensation of tension in neck and shoulders	32 (33)	143 (49)	<0.01
Pain all over the body	20 (20)	124 (43)	<0.01
Loss of appetite	16 (16)	81 (28)	0.024

## Discussion

Current literature shows ample evidence of great degrees of stress in medical students due to lengthy syllabi and challenging examinations (6, 16–18). However, there is lack of literature on medical education that specifically focuses on the impact of grading and assessment systems on academic stress in medical students. Medical students have identified studies and examinations as major stressors and their impact on their physical and mental well-being and academic performance (28).

In the present study, we used the WTAS to gauge the degree of anxiety and consequent impairments related to academic examinations. Students enrolled in the GPA system had a significantly higher mean score on the WTAS compared to students in the pass/fail system (2.8 vs. 2.4; *p*=0.01) as well as a greater percentage of students scoring on the higher side of the scale (40% vs. 23%; *p*=0.03), indicating a greater degree of examination-related anxiety (or test anxiety) in students enrolled in the GPA assessment system. The PSS was used to determine the perception of stress among the medical students enrolled in both assessment systems. Students assessed under the GPA system had a significantly higher mean score on the PSS in comparison to those in the pass/fail system (20.3 vs. 17.0; *p*<0.01) as well as a greater proportion of students having ‘above average’ stress (77% vs. 57%; *p*<0.01), indicating a greater degree of stress in GPA students. A study conducted at the Mayo Medical School (29) demonstrated that implementing a pass/fail assessment system in the first year of medical school led to decreased levels of perceived stress in students than students assessed under the five-interval grading system. Although this finding was similar to our results according to the PSS, no significant differences were found in test-taking anxiety in both groups, although it should be noted that students in the pass/fail system had better mood and greater group cohesion. Similarly, another study (30) conducted at the School of Medicine, University of Virginia, showed that applying a pass/fail assessment system in the first 2 years of medical school resulted in greater psychological well-being, as well as satisfaction with quality of education and personal lives. A systematic review by Spring et al. (28) showed that pass/fail assessment systems were associated with reduced perceived stress and anxiety among medical students and greater group cohesion, as well as satisfaction with academic performance and quality of education. However, it should be noted that some studies in this review stated that reduced levels of stress were only short-term and eventually became similar to stress levels in students in grade-tiered assessment systems in senior years of study. One study (31) involving several U.S. medical schools showed that students assessed under a grade-tiered assessment system of three or more categories were more likely to experience high levels of stress and emotional exhaustion. Not only this, students in the grade-tiered systems were also at an increased likelihood of having burnout as well as seriously considering the option of dropping out of medical school altogether.

It has been reported that females show a greater expression of personal emotions. This holds true for both positive emotions (love, happiness, and warmth, etc.) as well as negative emotions (fear, anxiety, etc.) (16, 32–34). In our study, there was a significantly larger number of female participants from the GPA system (85% vs. 59%; *p*<0.01). This large difference in gender proportions between the two assessment systems may be a possible reason why we observed higher levels of test anxiety and perceived stress in the GPA system in comparison to the pass/fail system. However, it should be noted that medical schools in Pakistan usually have a higher percentage of female students; hence this gives a more accurate representation of the medical student population in medical schools across the country. Some studies have correlated female gender with stress in students. A study conducted in a Swedish medical school showed female students to have a greater degree of anxiety, stress, and depressive symptoms (16). However, some studies oppose the associations of female gender with stress levels. For example, a study assessing the prevalence of stress in students at a medical school in India showed that there was no significant effect of gender on the stress levels (35).

Headaches were the most frequently reported physical symptoms associated with stress in both assessment systems. A larger percentage of students in the GPA system reported to have headaches associated with stress (74% vs. 50%, *p*<0.01). The link between stress and headaches is a well-established fact, stress being a precipitating factor for both acute and chronic episodes of headaches (36). In one study, approximately 90% of study participants attributed headaches as directly related to sources of stress in their respective lives (37); however, there are reports of no significant differences in the number of episodes of headaches in people with high stress and people with normal/low stress (38). Sapolsky (39) described the neuro-endocrinological responses to stress. The cumulative release of cortisol pulses in a state of physical and psychological stress (40) is a major cause of the sensation of aches and pain all over the body. In our study, 20% of the students in the pass/fail system reported to experience aches and pains all over the body as compared to 43% students in the GPA system (*p*<0.01).

The GPA assessment system is thought to encourage students to take rigorous classes, allowing school systems to offer more challenging courses with standardised grading and enabling students to compete on a uniform platform. Studies highlight the GPA system to be associated with study success, career development, and achievement (41). In contrast, the pass/fail system was thought to blur distinctions between students of different academic abilities and diminish students’ motivation for academic distinction (42). However, this point was disproven in a study by Robins et al. (43) in which they demonstrated that there was no decrease in the student motivation to learn, as students performed as well as they did before the implementation of the pass/fail assessment system. Nor was there any indication that students learned only enough to pass in the pass/fail system. Similarly, another study (30) showed the same observation that the pass/fail system did not reduce student motivation or performance in medical school or licensing examinations. Not only this, but the pass/fail system has been found to be associated with a higher degree of satisfaction with the standard of education and academic performance than grade-tiered assessment systems (28, 30). Our findings were consistent with this observation, where students lying in the completely satisfied groups in regards to academic performance were more in the pass/fail system than the GPA system. The proportion of students not satisfied or only moderately satisfied with their academic performance was greater in the GPA assessment system. However, the success of residency placement and postgraduate education and career development was not assessed for students enrolled under the two assessment systems in this study. This may be an area of future research interest in Pakistan.

Our results highlight that students being assessed under a pass/fail system have a comparatively less stressful academic lifestyle than students in a GPA system. These findings can be applied during the process of selecting candidates for residency and postgraduate education programs and used to assess their performance in the long term. It is suggested that the residents coming from a grading-tiered systems (similar to GPA assessment system) of assessment in their undergraduate medical education may perform better than those who came from a pass/fail assessment system (44). The association between stress levels of students at undergraduate level and their performance at postgraduate level can form the basis of future research and exploration. Opponents to the implementation of the pass/fail system assert that the students’ probability of matching in the desired residency programs may be adversely affected as residency program directors favour grade-tiered systems because of the difficulty in distinguishing high achieving students from ‘just passing’ ones in the pass/fail system (28, 43). Most researchers, however, have reported that although a few program directors admitted to prefer the grade-tiered assessment systems when selecting students for residency programs (45), these results were only opinion based (28) and no further evidence is available to establish that these opinions were actually acted upon. In fact, studies have demonstrated that a change from grade-tiered assessment to pass/fail system did not have a significant effect on the success of medical students in residency matching and placement (30, 43), quality of residency programs, career development and the number of students who ranked in the top 15 residency programs (30, 45, 46) and licensing examination scores in the United States (43). However, this was not explored in our study and is a potential topic of future research interest in Pakistan. Another limitation of our study is the method of sampling. Convenient sampling does not completely eliminate the risk of selection bias and this could have had possible effects on our findings. Prospective studies with larger student samples assessing the level of academic stress separately in each pre-clinical year will provide better insight into this subject and is a potential approach to studying this aspect of undergraduate medical education.

## Conclusion

In this study, we evaluated the level of stress among undergraduate medical students using the WTAS and the PSS. The levels of stress, as indicated by the two scales, were compared between two groups: the GPA system and the pass/fail system. The findings of this study can guide us to reform the medical curriculum and assessment system employed at the various medical colleges in Karachi, Pakistan, as well as the rest of the country. This would accomplish the aim of reducing the academic stress and exam anxiety in medical students while improving academic performance and having no negative effect on the level of motivation for academic excellence in medical students. To the best of our knowledge, a study comparing stress in two different assessment systems has not been conducted in our part of the world. So when it comes to formulating a grading system for medical students, it only seems reasonable that one which puts the students under less stress be preferred over the rest. A balance should therefore be reached between student well-being and academic outcomes when designing an academic assessment system. These findings indicate that governing bodies, such as the Higher Education Commission in Pakistan, which design and implement assessment systems and curricula in medical schools, revise the medical curriculum framework and employ a uniform assessment system for all the medical colleges under their supervision which should most positively impact the medical student's life by decreasing these stressors, allowing for better performance and outcome for the students in their professional careers. Our results show that the pass/fail assessment system accomplishes these objectives more efficiently than the GPA assessment system.

## Appendix 1

Learning and teaching system and assessment system at each medical institute

**Table:**

	Pass/fail system	GPA system
Teaching and learning system	Modular (organ system based) system	Modular (organ system based) system
Assessment system	• Modular examination (2–8 weekly) • Annual examination • Pass/fail/pass with honours grading	• Modular examination (2–8 weekly) • Semester examination (6 monthly) • GPA grading (4 interval grading)

## Appendix 2

Interpretation of Westside Test Anxiety Scale scores and scoring guideline and interpretation of Perceived Stress Scale

**Table 1:** Interpretation of Westside Test Anxiety Scale scores

Scores	Interpretation
1.0–1.9	Comfortably low test anxiety
2.0–2.5	Normal or average test anxiety
2.5–2.9	High normal test anxiety
3.0–3.4	Moderately high (some items rated 4=high)
3.5–3.9	High test anxiety (half or more of the items rated 4=high)
4.0–5.0	Extremely high anxiety (items rated 4=high and 5=extreme)

**Table 2:** Scoring guideline for the Perceived Stress Scale items

For questions 1, 2, 3, 6, 9, and 10 use the coding below to get the numerical score for each of the responses:				
0=Never	1=Almost never	2=Sometimes	3=Fairly often	4=Very often
For questions 4, 5, 7 and 8 use the reverse coding below to get the numerical score for each of your responses:				
4=Never	3=Almost never	2=Sometimes	1=Fairly often	0=Very often

**Table 3:** Interpretation of the Perceived stress scale scores

Total score	Perceived stress level is:	Health concern level
0–7	Much lower than average	Very low
8–11	Slightly lower than average	Low
12–15	Average	Average
16–20	Slightly higher than average	High
21 and over	Much higher than average	Very high
